# Supplementary material for: ΔFBA—Predicting metabolic flux alterations using genome-scale metabolic models and differential transcriptomic data
Source: PLoS Comput Biol. 2021 Nov 10;17(11):e1009589. doi: 10.1371/journal.pcbi.1009589 (PMC8608322; doi:10.1371/journal.pcbi.1009589)
Supplement: S1 Text — (PDF) [file pcbi.1009589.s001.pdf]

S1 Text

ΔFBA – Predicting metabolic flux alterations using genome-scale metabolic models and differential transcriptomic data

Sudharshan Ravi, Rudiyanto Gunawan

<u>Threshold Criteria for Minimum Flux Change Magnitudes</u>

The thresholds for the minimum magnitude of positive and negative flux difference, μ_i and η_i in Equations (4)-(7), respectively, are user-defined parameters. In the case studies, we set these thresholds a constant value ε, that is:

$$\mu_i = \eta_i = \varepsilon \quad \text{(S1)}$$

Thus, Δv_i will be greater than ε (default = 0.1% of the maximum flux bound) when $z_i^U = 1$. Correspondingly, Δv_i will be less than $-\varepsilon$ when $z_i^D = 1$. Below, we tested using a threshold that scales with the fold-change reaction expression $e_i^{P/C}$, such that:

$$\mu_i = \varepsilon e_i^{P/C} \quad \text{(S2)}$$

$$\eta_i = \varepsilon / e_i^{P/C} \quad \text{(S3)}$$

In the above equations, ε is again set to a constant value (default = 0.1% of the maximum flux bound). In this case, when $z_i^U = 1$, Δv_i will be greater than a value that is scaled proportional to the fold change of upregulated reaction expression. Analogously, when $z_i^D = 1$, Δv_i will take a negative value lower than a value that is proportional to the fold-change of the downregulated reaction expression. Such a scaling introduces a more stringent constraint on Δv_i.

We tested the thresholds prescribed by Equations (S2)-(S3) above for the Ishii *et al.* study [1], and compared the predicted flux changes $\Delta\boldsymbol{v}$ with those obtained with a constant threshold in Equation (S1). The more stringent threshold that scales proportionally with the fold changes of the reaction expression produced similar flux differences $\Delta\boldsymbol{v}$ to the constant threshold, as shown in **S1 Fig**. The differences in the prediction performance of ΔFBA were only marginal (Mean correlation coefficient (ρ) using Equation (S1) threshold = 0.61 and using Equations (S2)-(S3) threshold = 0.57; Mean directional accuracy using Equation (S1) threshold = 0.49 and using Equations (S2)-(S3) threshold = 0.49; Mean NRMSE using Equation (S1) threshold = 0.14 and using Equations (S2)-(S3) threshold = 0.16).

References

1. Ishii N, Nakahigashi K, Baba T, Robert M, Soga T, Kanai A, et al. Multiple high-throughput analyses monitor the response of E. coli to perturbations. 2007;316. doi:10.1126/science.1132067
