# Supplementary figures and images for: ΔFBA—Predicting metabolic flux alterations using genome-scale metabolic models and differential transcriptomic data
Source: PLoS Comput Biol. 2021 Nov 10;17(11):e1009589. doi: 10.1371/journal.pcbi.1009589 (PMC8608322; doi:10.1371/journal.pcbi.1009589)

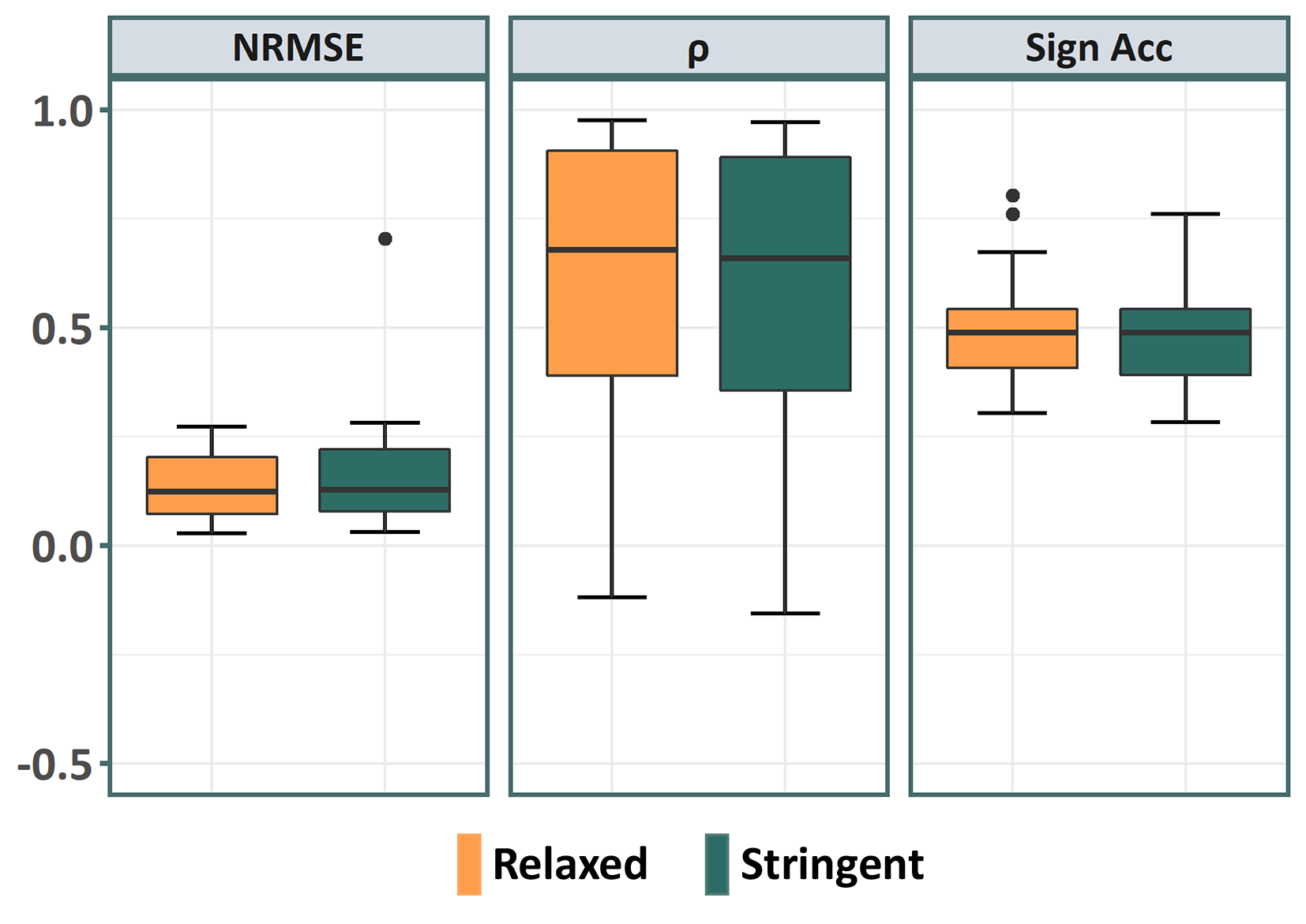

Supplement: S1 Fig — (Left) Normalized root mean square error (NRMSE), (Middle) uncentered Pearson’s Correlation Coefficient (ρ), (Right) Sign accuracy (Sign Acc) between the predicted flux difference and the measured flux change. The error bars show standard deviation across for 4 dilution rates (0.1, 0.4, 0.5, and 0.7 hours−1) and 24 single-gene deletions (galM, glk, pgm, pgi, pfkA, pfkB, fbp, fbaB, gapC, gpmA, gpmB, pykA, pykF, ppsA, zwf, pgl, gnd, rpe, rpiA, rpiB, tktA, tktB, talA, and talB). The difference in performance is not statistically significant. (TIF) [file pcbi.1009589.s002.tif]

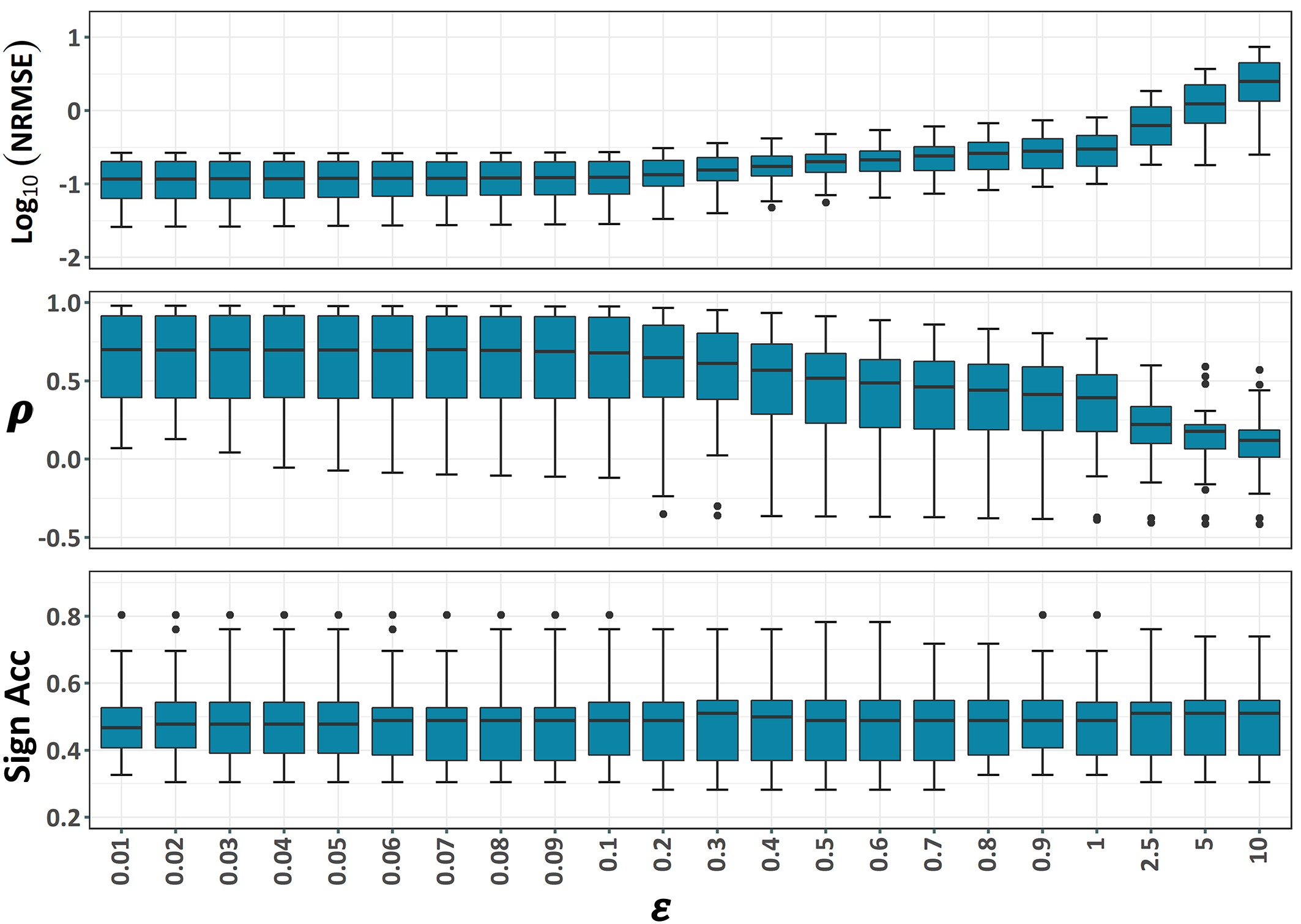

Supplement: S2 Fig — The default ε is 0.1% of the largest flux in the metabolic model under growth maximization and parsimony criteria. The error bars show standard deviation across flux difference predictions for 4 dilution rates and 24 single-gene deletions. The result indicates that the performance of ΔFBA is relatively insensitive to ε between 0.01% and 1%. (TIF) [file pcbi.1009589.s003.tif]

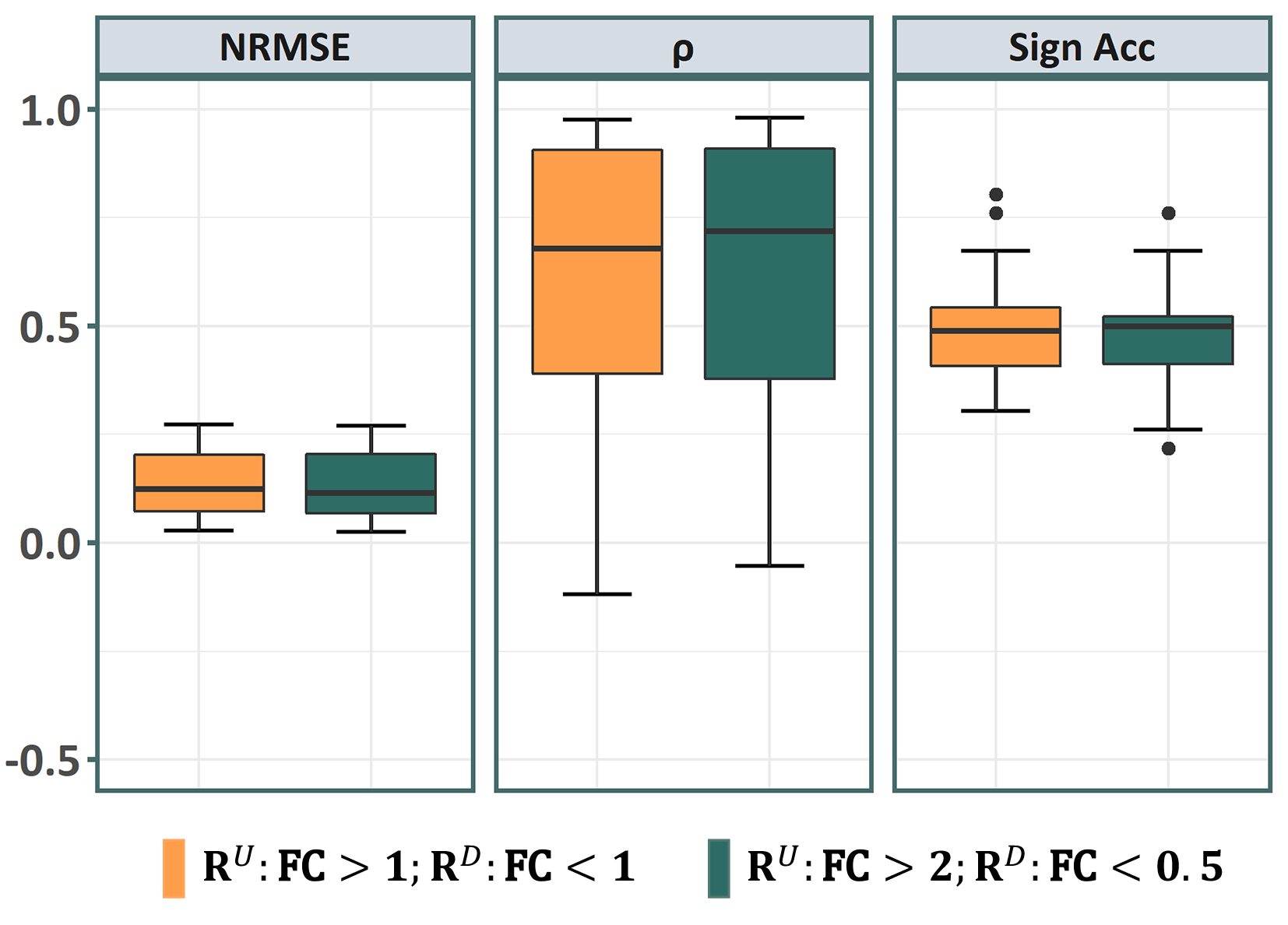

Supplement: S3 Fig — The default FC cut-off is 1. The error bars show standard deviation across for 4 dilution rates and 24 single-gene deletions in the Ishii et al. study [35]. The difference in performance is not statistically significant (Mean NRMSE—FC cutoff of 1 = 0.14, FC cutoff of 2 = 0.13; Mean ρ—FC cutoff of 1 = 0.61, FC cutoff of 2 = 0.63; Mean sign accuracy—FC cutoff of 1 = 0.49, FC cutoff of 2 = 0.48). (TIF) [file pcbi.1009589.s004.tif]

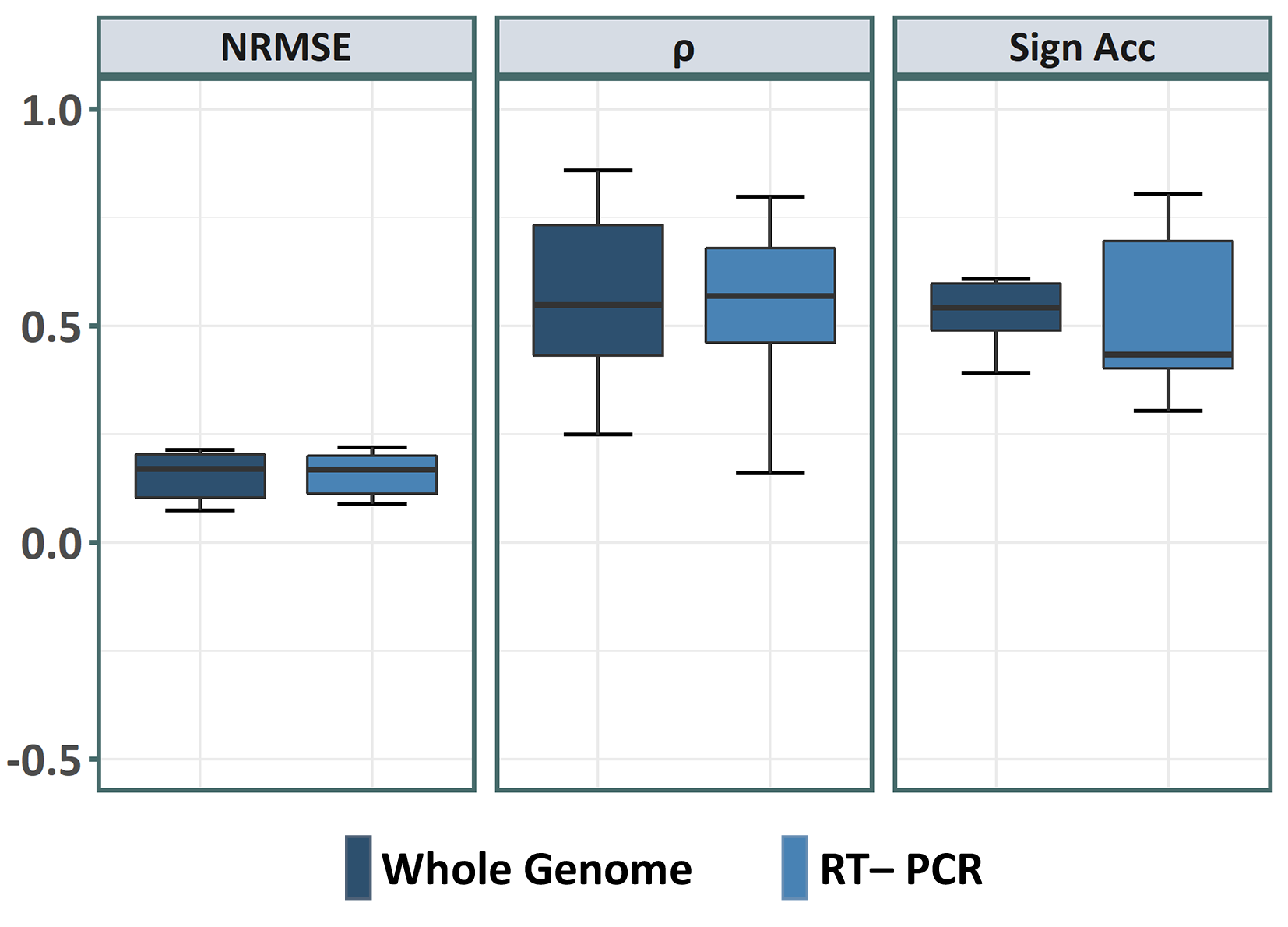

Supplement: S4 Fig — Directional (Sign Accuracy) agreement and uncentered Pearson’s Correlation Coefficient (ρ) between the predicted and measured flux differences have little difference between the incorporation of the two transcriptomic sources using ΔFBA (Mean NRMSE: whole-genome = 0.15, RT-PCR = 0.16; Mean ρ: whole-genome = 0.57; RT-PCR = 0.54; Mean sign accuracy: whole-genome = 0.53, RT-PCR = 0.53). (TIF) [file pcbi.1009589.s005.tif]

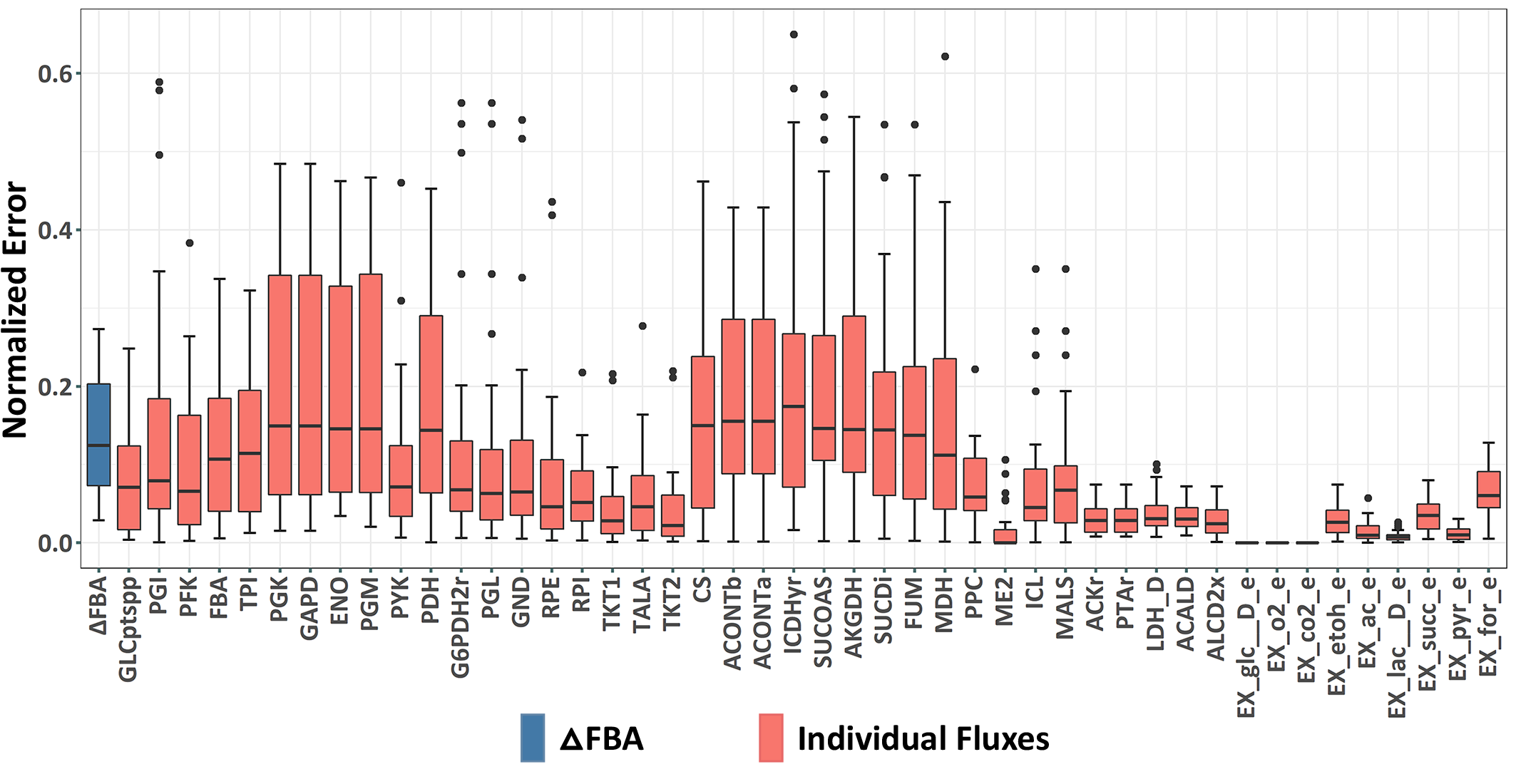

Supplement: S5 Fig — The NRMSE for the full flux differences is shown in blue (leftmost box plot). The remaining box plots in red show the distribution of the normalized error (NE) for each flux i: NEi=(ΔviM−Δvi*)2ΔvmaxM−ΔvminM, across 28 conditions (4 dilution rates and 24 single-gene deletions). (TIF) [file pcbi.1009589.s006.tif]
