# Supplementary material for: ΔFBA—Predicting metabolic flux alterations using genome-scale metabolic models and differential transcriptomic data
Source: PLoS Comput Biol. 2021 Nov 10;17(11):e1009589. doi: 10.1371/journal.pcbi.1009589 (PMC8608322; doi:10.1371/journal.pcbi.1009589)
Supplement: Attachment — Submitted filename: Response to reviewers.pdf [file pcbi.1009589.s007.pdf]

Response to Reviewers

Reviewer #1: *The Authors propose DeltaFBA, a method to identify flux changes using transcriptomic differential data. Although the idea seems novel and timely, there are however some questions that must be addressed:*

1) Method Formulation

The section where the MILP problem is presented is not sufficiently clear. Some definitions are lacking, and there are several typos, which hampers the correct interpretation of the proposed method and ultimately may obscure the rationale of DeltaFBA.

*The Authors should check that *all* of the variables are formally defined, namely concerning the dimensionality and context.*

Response:
We would like to thank the reviewer for valuable comments, questions, and suggestions. We have revised our manuscript following recommendations from the reviewer as well as the other two reviewers. Major revisions are highlighted in red in the revised manuscript. In response to the reviewer comment above, we have carefully gone through the method formulation and ensure that all variables are defined formally, including their dimensionality, in their first usage.

In the revised manuscript, we reorganized the text in Method Formulation section for better clarity. The mathematical formulation of ΔFBA is now followed by explanation about the variables and constraints. Any user-defined inputs are presented together in the next paragraph.

Comment:
- L122 - DeltaFBA is not enforcing the equation since S.delta_v=0 derives from considering that each C and P states are stationary.

Response:
The flux balance equation for the flux difference $\mathbf{\Delta v}$, specifically $\mathbf{S\Delta v} = 0$, is an equality constraint in the constrained mixed-integer linear programming (see Equation (2)). The flux difference $\mathbf{\Delta v}$ produced by ΔFBA is constrained to satisfy this equation.

Comment:
- L128 – S=0?

Response:
We apologize for the mistake. This has now been fixed.

Comment:

- Eq. 1 – max missing? Where is delta_v in the equation?

Response:
This has now been fixed. We apologize for the confusion. We removed $\boldsymbol{\Delta v}$ from the optimization variable since the vector is not directly optimized in the constrained MILP in Equation (1). The L2 norm optimization in the second step produces the prediction for $\boldsymbol{\Delta v}$, as described in the last part of Method Formulation.

Comment:
- The formal definition of z is lacking. Where is z0 in the equation? What is the dimension of z? In Eq.(3), it seems n. That affects the comprehension of the MILP problem.

Response:
The explanation for the variables involved in the constrained MILP, including $\boldsymbol{z}^0$, can now be found immediately after the mathematical formulation for improved clarity. Note that $\boldsymbol{z}^0$ is not being optimized. Rather, it serves two purposes: (1) to force certain flux difference to 0 if desired by a user, and (2) to force one of the reaction halves from a reversible reaction to have zero difference—that is, we only allow one direction of a reversible reaction to have non-zero flux difference.

Comment:
- The parameters wi are always set to values larger or equal to zero?

Response
Indeed, the weights are non-negative. We have clarified this in Line 169.

Comment:
2) Choice of the parameters

How can one choose the parameters in an unbiased way? I suppose the values wi, n, vmax, vmin, and epsilon influence the outcome. It would be interesting to more deeply analyze the sensitivity of the results to the choice of these parameters.

Response:
The upper and lower bounds for the flux differences are set based on experimental data or the corresponding upper and lower bounds for fluxes for the individual conditions (C and P). This procedure is described in Lines 176-179 and Equations (12)-(13). In the case studies, we followed the published flux bounds with the GEMs for *E. coli* and human *iMyocytes.*

The weights have default values of 1—that is, all reactions in the sets of upregulated and downregulated reactions have the same importance. In the Ishii *et al. E. coli* case study, we have knowledge of the specific reaction that is knocked out. Here, we set w_j^D for the knocked-out reaction to 10. We noted that in this case study, using the default $w_j^D = 1$ also produced the same solution for the knocked-out reaction—that is, we still obtained the

same negative flux difference for the deleted reaction. Nevertheless, we wish to stress that these weights are user defined inputs and some trial-and-error maybe needed to obtain the desired flux differences by adjusting the weights and rerunning the method.

The threshold epsilon can indeed affect the accuracy of the result. We have recommended to use 0.1% of the largest flux bound from the two conditions (control and perturbed). In the revised Supplementary Material, we demonstrated that the accuracy of flux differences by ΔFBA stays relatively high with epsilon between 0.01 to 10% (see Supplementary Figure S2).

We are unsure what the reviewer meant with the parameter *n* as this is the length of metabolic flux vector. If the reviewer is referring to parameter M in Equations (4)-(9), then this variable is related to the "Big M" strategy that is used in simplex algorithm for solving linear programming. In this case, as commonly done, M is set to a large value (default 10^5). We have clarified this in Lines 162-164.

Comment:
3) Correspondence between reactions and enzymes
Does DeltaFBA assume a one-to-one correspondence between reactions and enzymes? It seems that it is the case since the delta_v agrees with the direction of the gene expression changes (L.143), and the sets R^U and R^D are defined a priori. Can the authors comment on what happen when there are many-to-many relationships?

Response:
The set of up- and down-regulated reactions are user-defined inputs. In the case studies, we have used the Gene-Protein-Reaction (GPR) Max/Min rule to map differential gene expression data to reaction expression. In response the reviewer's comment, we added a new section called Gene-Protein-Reaction Mapping detailing the GPR Max/Min rule, immediately after Method Formulation.

Regarding the sets of up- and down-regulated reactions, we had used fold-change cutoffs for reaction expression in our case studies. Certainly, the prediction of flux differences by ΔFBA depends on these user-defined inputs. But, as we have now shown for Ishii *et al.* study in Supplementary Figure S3, the flux prediction accuracy did not change markedly by increasing the fold-change cutoff from 1 to 2 for upregulation reactions, and from 1 to 0.5 for downregulated reactions.

Comment:
Does the fact that "Only a small fraction of variation in the measured flux ratios can be explained by the fold-change in reaction expressions..." (L.281) affect the overall rationale of the approach? Please comment.

Response:
Our approach is driven by two key assumptions: (1) that the flux differences satisfy the flux balance equation—an assumption that follows directly from steady-state flux balance in

control and perturbed conditions, and (2) that the flux differences should be maximally consistent with the gene expression. The second assumption is captured by the constrained MILP formulation. Note that we allow for inconsistency between differential gene expression and flux difference—that is, gene expression maybe downregulated, but the flux difference is positive—but this is being minimized. We have clarified this point in Lines 334-341.

Comment:
4) Performance evaluation
How are the differences between the measured fluxes and the predicted fluxes distributed? The evaluation measures proposed (L.254) seem not to take individual discrepancies into account; for example, are there any outliers that may be skewing the results? Or, in other words, is it possible to interpret for which fluxes the methods most fail (or are able to better guess the correct value)?

Response:
Thank you for this suggestion. In Supplementary Figure S5, we presented the prediction accuracy for individual reactions for which there were ground truth ^{13}C flux data. We noted that reactions that form futile cycles are associated with higher errors, which is to be expected, since such cycles are associated with flux degeneracy in FBA. We have mentioned the above point in Lines 361-365.

Minor Comment 1:
Correct "Equation Error! Reference not found" (L.162, L.166).

Response:
This has now been corrected. We apologize for these errors, which might have been introduced during PDF conversion.

Minor Comment 2:
L.289 – The 4 dilution rates and 24 single-gene deletions are not combinatorial – are the changes combinatorial? Or have they done all-at-once?

Response:
The perturbations were implemented one at a time. We have clarified this now in Line 347.

Reviewer #2: *Ravi and Gunawan present the manuscript title "ΔFBA – Predicting metabolic flux alterations using genome-scale metabolic models and differential transcriptomic data" describe an alternative tool to predict metabolic flux alteration by impose additional constraints derived from transcriptome data. The author demonstrated the developed tool and assessed the performance in various experiments of E.coli and human skeleton muscle system.*

Response:
We would like to thank the reviewer for his/her comments on our work. We have revised our manuscript, where the key revisions are highlighted in red, based on the comments provided by the reviewer and the other two reviewers.

Comment:
- Abstract need to be improve by clearer point out the key contributions of the work.

Response:
We have revised the abstract and author summary for better clarity on the key contributions of our work.

Comment:
- There re "Error! Reference source not found" in the manuscript that need to be fixed.

Response:
We apologize for these errors, which were possibly introduced during conversion to PDF, which we have now checked and corrected in the revised manuscript.

Comment:
- It is not clear (not be mentioned) how statistical Pvalue which is the important parameter for considering the usage of fold changes. If the fold changes have high pvalues, we cannot use the changes as addition constrains in flux calculation due to uncertainty.

Response:
Wherever appropriate, we had filtered out genes with fold-change expression beyond a statistical significance (FDR > 0.05). These are now mentioned in Lines 252-253 and Lines 266-267. But, the decision to include reactions in the sets of up- and down-regulated reactions is done based on the fold-change reaction expression. The implication of statistical significance of differential gene expression on the regulation of metabolic fluxes is unclear. More specifically, there is little evidence (either in the literature or in our own experience) that the more statistically significant fold-change (lower *p*-value) in gene expression is connected to more significant changes in the corresponding metabolic fluxes. In our work, we opted for a more direct relationship between fold-change in gene expression and changes in the fluxes. More specifically, in the case studies, we chose to use simple cutoffs to prescribe the up- and down-regulated reactions—using either a fixed fold-change (FC) reaction expression cutoff or percentiles. In our experience, this approach worked relatively

well, and the comparison with other methods further gave support to this approach. We also tested using a different fold-change cutoff (FC > 2 for upregulation and FC < 0.5 for downregulation) and the accuracy of the flux difference predictions remained approximately the same (see Supplementary Figure S3).

At the same time, we wish to stress that the sets of up- and down-regulated reactions are user-defined inputs. Users are of course free to use other approaches, including statistical (p-value) cutoffs as suggested by the reviewer, to define these sets for their datasets and their study.

Comment:
- The gold standard of metabolic flux is C13 labeling experiment which include in some datasets that the author used in case study. I recommend comparing the results from dFBA with C13 flux in details.

Response:
Indeed, as the reviewer mentioned, the performance assessment in this work was all done by comparing the prediction of flux differences with the ^{13}C flux data, specifically in the Ishii *et al.* and Gerosa *et al.* case studies. The results in Figures 1 and 2 were based on using these ^{13}C data as the ground truth.

Comment:
- It is not clear for me how performance evaluations were performed. What is the ground truth that author use to estimate performance?

Response:
We apologize for the lack of clarity about the ground truth. As noted in our response above, we used ^{13}C metabolic flux data from Ishiii *et al.* and Gerosa *et al.* studies for assessing the performance of ΔFBA. As described in Materials and Methods, we used three different metrics for performance: normalized root mean square of error (NRMSE), correlation, and sign accuracy. We have now clarified this point in Lines 302-303.

Comment:
- Based on the report, dFBA have the accuracy <= 0.7, Is it considered good? ROC analysis should be performed and provide in detail for the readers.

Response:
ROC is typically used to assess accuracy for a binary classification problem, which does not directly apply to our work. In this work, we are interested in estimating the values of flux differences. Nevertheless, one of the performance metrics is the sign accuracy, which can be thought as a measure of accuracy in a ternary classification problem, specifically positive, negative, and zero flux differences. The sign accuracy is computed as the fraction of accurate predictions relative to the total number of predictions.

Comment:
- I suggest the authors perform more case studies in yeast system that have a lot of data of transcriptome and C13 flux (some studies provided).

Respond:
We had difficulty in finding studies that report both transcriptome and C13 flux data together. Also, various studies use different yeast strains that do not directly correspond to the available genome-scale metabolic model for yeast. For these reasons, we had not included any case studies using yeast data. But, during the revision, we added eight additional methods to which we compared ΔFBA. The results are presented in the revised Figure 1.

Comment:
- The manuscript lack of sensitivity analysis that need for new developed tool like dFBA.

Response:
In response to the reviewer's comment, we performed sensitivity assessment of ΔFBA with respect to the epsilon threshold and the fold-chance cutoff for specifying the up- and down-regulated reactions. The results can be found in Supplementary Figures S2-S3. In general, the performance of ΔFBA does not depend sensitively on these parameters.

Comment:
- For transparency and reproducibility, the author needs to provide all computational codes used for the case studies presented in the manuscript in the GitHub for the reader.

Response:
The codes and the full results of the case studies are now available on the GitHub page for ΔFBA (http://www.github.com/cabsel/deltafba).

Reviewer #3*: In this work, Ravi and Gunawan present a new method to integrate transcriptome data and genome-scale model to interpret the rewiring in metabolism non-intuitively. While this work is under the scope of PLOS Comp Bio and presents substantially novel and useful algorithm, its superiority and applicability over existing algorithms should be clearly explained than now. Some of my major comments as follows:*

Response:
We would like to thank the reviewer for the comments on our work. We have revised our manuscript following recommendations by the reviewer and the other two reviewers. The key revisions are highlighted in red. Below we provide our detailed response to the reviewer's comments.

Comment 1:
I think the authors have not thoroughly compared their new method with all the previously existing ones. While they have compared it only with REMI, it is essential to compare it with many other similar algorithms, i.e., integrating differential gene expression data with GEM. While the authors have cited the MOONMIN, Zhu et al as other methods in this category, I'm aware of several others such as tFBA (van Berlo, 2011), MADE (Jensen and Papin 2011), AdaM (Topfer 2012) and GX–FBA (Navid and Elmaas, 2012). Therefore, I would like the authors to comprehensively review the literature in this regard and compare their methods with previous ones, showcasing the strengths of their algorithm over previous ones.

Response:
Thank you for the suggestion. We had originally compared ΔFBA to REMI since REMI was the most recent algorithm developed for predicting changes in metabolic fluxes using differential expressions.

In response to the reviewer's comment, we included more methods in our comparison. More specifically, we followed a previous systematic comparison of FBA methods with transcriptome integration by Machado and Herrgard (2014), and added 8 additional methods from this study, including some of the methods mentioned by the reviewer. The revised Figure 1 shows that ΔFBA significantly outperforms all of these methods in terms of NRMSE and Pearson correlation, while all methods have comparable sign accuracy. For the sign accuracy, we noted that ~18% of the ground truth flux difference values are 0. Most methods, including ΔFBA, do not generally produce zero flux difference—GIMME produced the most zero flux differences because the method involves removal of reactions from the metabolic network.

Comment 2:
In the implementation, it is unclear how the authors deduced up-/down-regulated reactions from up-/down-regulated genes. There exists multiple scenarios which is not straightforward – a reaction with multiple isoenzymes could have different isozymes up-/down-regulated. Similar cases could exist with gene subunits. It is important for authors to provide more details in this regard.

Response:
We apologize for the lack of clarity in the mapping from gene expression to reaction expression. In the revised manuscript, we provided more detail on the GPR Max/Min rule that we had used in a new section called **Gene-Protein-Reaction Mapping** (Lines 208-224), which can be found immediately after **Method Formulation** in **Materials and Methods**.

Comment 3:
Integration of gene expression data with GEM is a one of widely researched topic in this field. So far, two methods have been predominantly proposed – using transcriptome data in the form of differentially expressed genes as used here and the use of a threshold to transform gene expression data onto binary form and then use it. It may be better to authors to comment on these two approaches in their paper and provide how this method complements and/or outperforms the other. I raise this point because, it is well known fact that the transcriptional regulation of metabolic flux is not well correlated and it is good to know which of these methods could capture this well.

Response:
Thank you for this suggestion. We have expanded the discussion about the combination of our method that uses differential gene expression with other methods that generate single-condition flux predictions (see Lines 478-495). We noted that Machado and Herrgard demonstrated in their systematic comparative study that parsimonious FBA often works better than methods that rely on gene expression data. In any case, once the metabolic fluxes for the reference condition is determined (and ideally fully validated), ΔFBA can be used to generate flux differences and with the reference fluxes, to predict fluxes for the perturbed condition. Note that for many different gene expression profiling technologies and because of other technical and biological reasons, relative expressions are more reliable than absolute expression data.

Comment 4:
Several equations in the paper were missing due to formatting error making it further difficult to evaluate the presented algorithm.

Response:
We apologize for these errors, which may have been introduced during a previous PDF conversion. We will ensure such errors do not appear in the revised manuscript submission.

Comment 5:
It is good that the authors have provided the code in GitHub, which helps its reproduction/implementation.

Response:
The codes and the full results of the case studies are now available on the GitHub page for ΔFBA (http://www.github.com/cabsel/deltafba).
